# Brief Extracts from the Proceedings of the Pennsylvania Association of Dental Surgeons

**Published:** 1856-10

**Authors:** 


### ARTICLE VII.

### *Brief Extracts from the Proceedings of the Pennsylvania Association of Dental Surgeons.*

*Meeting of Feb. 3d.*—Dr. Daniel Neal addressed the Association at considerable length on the subject of "Examination of the Mouth and Extirpation of Decay." The well known practical character of the Dr. will give an idea of the nature of his remarks.

In the course of the remarks, allusion was made to a great want of thoroughness constantly manifesting itself in the performance of dental operations, Dr. Neal affirming that in most cases where an operation on the teeth was pronounced completed, proper instruments would discover quite as many cavities as had already been treated. Exhibited to the members instruments employed by himself in examinations, which certainly seem capable of discovering the most minute cavity. Also suggested, in reference to the extirpation of sensitive dentine, that among the best obtunders should rank sharp instruments. Alluded to his manner of removing the dead bone, the care oftentimes found necessary to prevent inflammation of the pulp, presenting, indeed, an aggregate of important suggestions, which must have demonstrated to every member the advantages of dental association.

*Special Meeting, Feb.* 19.—The question for debate was "Alveolar Abscess."

Dr. Harris remarked—the subject was an interesting one; considered it imperfectly understood. Alluded to views entertained by different authors. Mr. Flourens maintaining that the internal periosteum is but a dipping through the foramen of the external, others hold such a view as a great error. If the first was true, then we had the same membrane performing a two-fold duty—secreting at one point, absorbing at another. If the reverse was true, then we might puzzle ourselves to explain the close existing sympathy.

The peculiar formation of the pus bag, the effects of so abnormal an agent on the surrounding living tissue—particularly its effect upon the particular organ involved—these, and the many collaterals could not be thought to be investigated without great accruing benefits, both to ourselves and to those whose sufferings we are called on to relieve.

Understanding the pathology of the disease, efforts and labor should be made in a hygienic direction; for, when it is considered the dependence of a tooth for health is on the vascular supply yielded through this membrane; and when, further, it is considered that it is not an improbable thing that it is the only source of tooth life, (and as, indeed, daily experience seems to prove,) it may be concluded that periostitis has so far progressed as to sever the attachment, the tooth is beyond any remedy of our pharmacopœia. For his own part, believed this to be the case, and would like to see the discussion take the direction of causes and prevention.

Prof. Buckingham.—As the result of, first, simple vascular excitement, and, second, inflammation running into gangrene, remarked that we had the disease known as periostitis once thoroughly fixed. Inclined with Dr. Harris to the belief that the teeth affected is beyond redemption. Suggested that it might remain in the mouth, and in individuals of certain temperaments, give service for years, but that it is a devitalized tissue, remaining in its position alone, as it were, through favor. Agreed that research should extend rather in the hygienic direction, as suggested, and, so far as its dento-manipulative causes were concerned, would offer for what they might be worth, his mode of procedure, when attempting the destruction of the pulp of a tooth, in order to prevent the too often attendant evil. Esteemed that he had been most fortunate, and his source of good fortune might be presented in very few words. After treatment of the pulp, always plugged up his cavity with some substance saturated with creosote. Had found that creosote, through its alterative or stimulant properties, acted the good Samaritan's part. In the treatment of abscess used morphia, plugging up the cavity so that on the occurrence of trouble, the patient could remove the cotton or whatever it was composed the plug. The Dr's remarks were extended to some length.

Dr. Daniel Neal.—This hygienic seems the proper direction in which to employ our attention, for, have we no disease, we want no remedy, and the quaint saying of poor Richard "that an ounce of prevention is worth a pound of cure," is here peculiarly applicable, or at least it so strikes us. Was forced from a long series of observations, to believe that the dentist held generally the key to this formidable disease. The contact of gold with a delicate membrane, from its conducting facility, opened the way to it in one direction; leaving diseased matter in a cavity led to it in another direction, and so on, many other excitants of which, was it necessary, mention might be made, and over which the dentist holds supervision.

Cleanliness, he remarked, was next to godliness; certainly, in dental therapeutics, was synonymous with salvation. In treatment, and particularly where a nerve cavity was concerned, exerted himself to the observance of this law. Was tempted, indeed, to believe it the *sine qua non.*

When, however, a case of alveolar abscess presented, his treatment, in part, was about the mode pursued for its prevention. Removed the decomposed animal tissue, getting as near the apex with the excavator as possible, taking away every thing causing fetor, and, through the medium of the syringe, endeavored to complete and perfect the purifying process. Thought that no abscess, however severe or alarming, should throw the dentist from his equilibrium. Extracting instruments were the very last in his pharmacopœia. Believing that judicious treatment might often save, and that tooth life, like human life, was of sufficient consequence in most cases, to command every exertion.

The Dr. mentioned several very interesting cases of alveolar abscess which he had successfully treated. In one case (that of a lad) the tooth and parts were so affected as to discharge at least a thimbleful of pus, and ultimately, the tooth became so loosened as to have permitted its removal with the fingers. It being, by reason of its position, of much use to his patient, felt compelled to use every exertion to save it. Treated it with camphor and cleanliness, and in ten weeks had the satisfaction to find it sufficiently firm to allow of its being filled with gold, which operation was performed, and the tooth has since remained useful and entirely comfortable, a year having passed. Mentioned this last as an extreme case, and thought the members would agree with him, that if the disease thus progressed, was conquered, it was sufficient reason to warrant exertion in any and every case.

Prof. J. D. White coincided with the views of Dr. Neal. Urged the necessity of the purifying process. If such a law was more closely observed, thought that in more directions than this one, the dentist would have reason for self-congratulation. It was the foundation law of health, and without such a corner-stone, strength could not be given the edifice.

Prof. Flagg alluded to the causes of abscess. Has often remarked the disease as resulting from an imperfect dental operation, and particularly where, in the attempted filling with gold of nerve cavities, the canal has not been plugged. Another fertile source was the setting of pivot teeth. In this direction, his experience has been that where arsenic is used to destroy the nerve, alveolar abscess is an almost certain sequence. As regards the nature of the disease, viewed it as a deep-seated ulcer, and pursued similar treatment.

Prof. Parry.—Has had much trouble. Never yet found a remedy anything like universally successful. The most successful ever yet employed, is now using. Is beginning to rest strongly upon it, and has had the happiest results. Uses chloroform freely about the abscess. If able, injects both through the foramen and abscess. When able to employ it in this last way, feels very confident.

President Townsend.—Will Prof. Parry give us his theory of the action of the agent?

Prof. Parry.—It acts both as a stimulant and alterative; acts similarly to nitrate of silver on diseased flesh.

Dr. Harris.—As other gentlemen had given their mode of treatment when meeting with the disease, would offer that adopted as a general rule by himself. Endeavors, with injections of nitrate of silver, from 10 to 30 grains to the ounce of water, to break up the disease as in other parts is attempted with escharotics; when necessary, cuts away surrounding integuments to as near the seat of disease as may be done with safety, that he may the better inject; mentioned, among others, the case of a lady where three roots of the same tooth were involved; the palatial fang was discharging its matter into the cavity of the mouth, and the two buccals outwardly; removed all the diseased matter possible; conquered the disease with the nitrate of silver, as alluded to; the tooth of course was dead, but served the patient without further annoyance for a series of years. The Doctor, in the continuance of his remarks, viewed the pathology of the disease at length, and referred to several cases to which he applied the name idiopathic.

Prof. Arthur.—Alveolar abscess never results from causes entirely constitutional; had never met a case where the predisposition had not in some degree a local origin. Any particular point to be effected must have in itself some weakness rendering it less able to resist disease than its neighboring parts; did not rise, however, to discuss the question, but desired to offer some few remarks on its plainer causes. When a tooth receives a blow, depriving the pulp of vitality, the dead matter is absorbed through the tubuli, exciting periosteal inflammation. As a result, we are tolerably sure of alveolar abscess, if an immediate and proper treatment is not adopted—a treatment very simple in itself, and with which all, of course, are familiar, "that of opening into the pulp cavity and removing the matter," &c. In regard to alveolar abscess resulting from treatment of the pulp with arsenic, in ninety-nine cases out of the hundred would attribute it to the carelessness or unskillfulness of the operator; from close observation, was convinced that facts would bear him out in this assertion. Nerve cavities are, as a general thing, too hastily prepared, the filling of gold too quickly succeeding the arsenical application. To remove the body of a pulp from its chamber is generally an easily accomplished task, but it is in the canal of the roots that we have our trouble; and to fill over the dead matter there contained is, in many cases, the cause of disease. Such, indeed, is the susceptibility of the periosteum to disease, that the absorption of decomposed food allowed to remain in a nerve cavity, will excite inflammation; cited several cases illustrative of the various causes, which were possessed of much interest and bearing on the subject.

Prof. Buckingham.—If using nitrate of silver at all, would use it strong; it might discolor, yet inclined to believe the tooth would soon resolve back to its original color; would, however, as previously remarked, prefer the use of creosote, because it does the work of the silver, yet has none of its objections; as a vapor it penetrates through the foramen, exercising its caustic alterative and stimulating effects on the part involved, breaking up disease and exerting to healthy action. Concerning constitutional predispositions, if it is remembered that a part once truly inflamed is ever more liable to be attacked than other parts, we may have an explanation in part of systemic influences centering on some particular tooth of the thirty-two, or however many there may be in the mouth; as referring back, we may remember the tooth involved as having at some previous time been the seat of disease.

Prof. Arthur.—Abscess resulting from pivoting teeth will be explained by my previous position, as I incline to believe—the non-removal of the entire nerve—and under any circumstances while this is allowed to remain all treatment is useless. The Doctor alluded to cases of abscess he had met, in which the pulp seemed not in the least involved.—*Ib.*

